# Synthesizing and Reconstructing Missing Sensory Modalities in Behavioral Context Recognition

**DOI:** 10.3390/s18092967

**Published:** 2018-09-06

**Authors:** Aaqib Saeed, Tanir Ozcelebi, Johan Lukkien

**Affiliations:** Department of Mathematics and Computer Science, Eindhoven University of Technology, Eindhoven, The Netherlands; t.ozcelebi@tue.nl (T.O.); j.j.lukkien@tue.nl (J.L.)

**Keywords:** sensor analytics, human activity recognition, context detection, autoencoders, adversarial learning, imputation

## Abstract

Detection of human activities along with the associated context is of key importance for various application areas, including assisted living and well-being. To predict a user’s context in the daily-life situation a system needs to learn from multimodal data that are often imbalanced, and noisy with missing values. The model is likely to encounter missing sensors in real-life conditions as well (such as a user not wearing a smartwatch) and it fails to infer the context if any of the modalities used for training are missing. In this paper, we propose a method based on an adversarial autoencoder for handling missing sensory features and synthesizing realistic samples. We empirically demonstrate the capability of our method in comparison with classical approaches for filling in missing values on a large-scale activity recognition dataset collected in-the-wild. We develop a fully-connected classification network by extending an encoder and systematically evaluate its multi-label classification performance when several modalities are missing. Furthermore, we show class-conditional artificial data generation and its visual and quantitative analysis on context classification task; representing a strong generative power of adversarial autoencoders.

## 1. Introduction

The automatic recognition of human activities along with inferring the associated context is of great importance in several areas such as intelligent assistive technologies. A minute-to-minute understanding of person’s context can enable immediate support e.g., for elderly monitoring [1], timely interventions to overcome addictions [2], voluntary behavior adjustment for living a healthy lifestyle [3,4], coping with physical inactivity [5] and in industrial environments to improve workforce productivity [6]. The ubiquity of sophisticated sensors integrated into smartphones, smartwatches and fitness trackers provides an excellent opportunity to perform a human activity and behavior analysis as such devices have become an integral part of our daily lives [7]. However, context recognition in a real-life setting is very challenging due to the heterogeneity of sensors, variation in device usage, a different set of routines, and complex behavioral activities [8]. Concretely, to predict people’s behavior in their natural surroundings, a system must be able to learn from multimodal data sources (such as an accelerometer, audio, and location signals) that are often noisy with missing data. In reality, a system is likely to encounter missing modalities due to various reasons such as a user not wearing a smartwatch, a sensor malfunction or a user not granting permission to access specific data because of privacy concerns. Moreover, due to large individual differences, the training data could be highly imbalanced, with very few (sparse) labels for certain classes. Hence, for a context recognizer to perform well in unconstrained naturalistic conditions; it must handle missing data and class imbalance in a robust manner while learning from multimodal signals.

There are a variety of techniques available for dealing with missing data [9,10]. Some naive approaches are, mean substitution or simply discarding instances with missing values. In the former, replacing by average may lead to bias (inconsistency would arise e.g., if the number of missing values for different features are excessively unequal and vary over time) [9]. In the latter, removal leads to a substantial decrease in the number of samples (mostly labeled) that are otherwise available for learning. It can also introduce bias in the model’s output if data are not missing completely at random [10]. Similarly, principal component analysis (PCA) approach could be to utilize through inverse transformation on the reduced dimensions of the original data to restore lost features but the downside is PCA can only capture linear relationships. Another approach might be training a separate model for each modality, where the decision can be made on the basis of majority voting from the available signals. Though in this scheme, the distinct classifiers will fail to learn the correlation that may exist between different sensory modalities. Besides, this approach is inefficient as we have to train and manage a separate classifier for every modality available in the dataset.

An autoencoder is an unsupervised representation learning algorithm that reconstructs its own input usually from a noisy version, which can be seen as a form of regularization to avoid over-fitting [11]. Generally, the input is corrupted by adding a Gaussian noise, applying dropout [12] or randomly masking features as zeros [13]. The model is then trained to learn a latent representation that is robust to corruption and can reproduce clean samples from partially destroyed features. Therefore, denoising autoencoders can be utilized to tackle reconstruction, while learning discriminative representations for an end task e.g., context classification. Furthermore, the adversarial autoencoder (AAE) extends a typical autoencoder to make it a generative model that is able to produce synthetic data points by sampling from an arbitrarily chosen prior distribution. Here, a model is trained with dual losses–reconstruction objective and adversarial criterion to match the hidden code produced via the encoder to some prior distribution [14]. The decoder then acts as a deep generative model that maps the enforced prior distribution to the data distribution. We address the issues of missing data and augmenting synthetic samples with an AAE [15].

**Figure 1:**
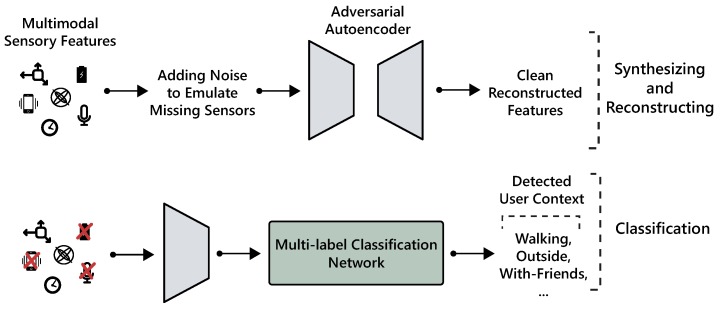
Overview of the proposed framework for robust context classification with missing sensory modalities.

In this paper, we present a framework based on AAE to reconstruct features that are likely to go missing all at once (as they are extracted from the same modality) and augment samples to enable synthetic data generation (see Figure 1). We demonstrate the representation learning capability of AAE through accurate reconstruction of missing values and supervised multi-label classification of behavioral context. In particular, we show AAE is able to provide a more faithful imputation as compared to techniques such as PCA and show strong predictive performance even in case of several missing modalities. We analyze the performance of the decoder trained with supervision enabling the model to generate class conditional artificial training data. Further, we show that AAE can be extended with additional layers to perform classification; hence leveraging the complete dataset including labeled and unlabeled instances. The primary contributions of this work are the following:

- Demonstration of a method to restore missing sensory modalities using an adversarial autoencoder.
- Systematic comparison with other techniques to impute lost data.
- Leveraging learned embedding and extending the autoencoder for multi-label context recognition.
- Generating synthetic multimodal data and its empirical evaluation through visual fidelity of samples and classification performance on a real test set.

We first address previous work on using autoencoders for representation learning and handling missing data in Section 2. Section 3 briefly reviews the large-scale real-world dataset utilized in this work for activity and context recognition. We then describe a practical methodology for restoring lost sensor data, generating synthetic samples, and learning a context classifier based on adversarial autoencoder in Section 4. In Section 5, we systematically examine the effect of missing modalities on context recognition model, show a comparison of different techniques, and evaluate the quality of synthetic data. We then provide a discussion of the results, highlight potential limitations and future improvements, followed by conclusions in Section 6.

## 2. Related Work

Previous work on behavior context recognition has evaluated fusing single-sensor [8] classifiers to handle missing input data, in addition to utilizing different combinations of sensors to develop models for each group [16]. However, these methods do not scale well to many sensors and may fail to learn correlations that exist between different modalities. Furthermore, restoration of missing features with imputation methods remains a non-trivial task as most procedures fail to account for uncertainty in the process. In the past, autoencoders have been successfully used for unsupervised feature learning in several domains thanks to their ability of learning complex, sparse and non-linear features [11]. To put this work into context, we review contemporary approaches to leveraging autoencoders for representation learning and handling missing input data.

Recent methods [17,18,19,20,21,22,23] on ubiquitous activity detection have effectually used the restricted Boltzmann machine, denoising and stacked autoencoders to get compressed feature representations that are useful for activity classification. These methods performed significantly better for learning discriminative latent representations from (partial) noisy input, that is not solely possible with traditional approaches. To the best of our knowledge, no earlier works in activity recognition domain explicitly addresses missing sensors problem except [24] that utilizes dropout [12] for this purpose. Nevertheless, several works in different areas have used autoencoders to interpolate missing data [25,26,27,28,29]. Thompson et al. [25] used contractive autoencoder for the restoration of missing sensor values and showed it is generally a non-expensive procedure for most data types. Similarly, Nelwamondo et al. [26] study the combination of an autoencoder and a genetic algorithm for an approximation of missing data that have inherent non-linear relationships.

In bioinformatics and healthcare community, denoising autoencoders (DAE) have been used to learn from imperfect data sources. Li et al. [30] used DAE for pre-training and decoding an incomplete electroencephalography to predict motor imagery classes. Likewise, Miotto et al. [31] applied DAE to produce compressed embedding of patients’ characteristics from a very large and noisy set of electronic health records. Their results showed major improvements over alternative feature learning strategies (such as PCA) for clinical prediction tasks. Furthermore, Beaulieu-Jones [28] systematically compared multiple imputation strategies with deep autoencoders on the clinical trial database and showed strong performance gains in disease progression predictions.

Autoencoders are also extensively used in affective computing to advance emotion recognition systems. Martinez et al. [32] applied a convolutional autoencoder on raw physiological signals to extract salient features for affect modeling of game players. In [33], autoencoders are utilized with transfer learning and domain adaption for disentangling emotions in speech. Similarly, Jaques et al. [29] developed a multimodal autoencoder for filling in missing sensor data for mood prediction in a real-world setting. Furthermore, DAE has been effectively demonstrated for rating prediction tasks in recommendation systems [34].

Generative adversarial network (GAN) [14] as a framework has shown tremendous power to produce realistic looking data samples, particularly images. It is also successfully applied in natural language processing domain to generate sequential data with a focus on discrete tokens [35]. Recently, they are also used in medical domains to produce electronic health records [36] and time-series data from an intensive care unit [37]. Makhzan et al. [15] combined classical autoencoders with GANs through the incorporation of adversarial loss to make them a generative model.

This makes AAE a suitable candidate for learning to reconstruct and synthesize with a unified model. However, to the best of our knowledge, no previous work has utilized them for synthesizing features extracted from multimodal time-series, specifically for context and activity recognition. Hence, models capable of successful reconstruction and generation of synthetic samples can help overcome the issues of noisy, imbalanced and access problems (due to sensitive nature) to the data, which ultimately helps downstream models to become more robust.

Our work is broadly inspired by efforts to jointly learn from multimodal data sources and it is similar to [29] in applied training strategy; though it utilizes an AAE for reconstruction, augmentation, and multi-label behavior context recognition. Besides, as opposed to [24], where a feed-forward classification model is directly trained with dropout [12] to handle missing modalities, here, the model first learn to reconstruct the missing features by employing both dropout and structured noise (see Section 4.4). Then, we extend this model with additional layers for multi-label classification through either directly exploiting the encoder or training a network from scratch with learned embedding. In this manner, the AAE based network will not just be able to reconstruct and classify but it can also be used for class conditional data augmentation.

## 3. ExtraSensory Dataset

We seek to learn a representation of context and activities by leveraging massive amounts of multimodal signals collected using smartphones and wearables. While there are a variety of open datasets available on the web, we choose to use *ExtraSensory Dataset* [8] because it was collected in a real-world environment when participants were busy with their daily routines. It provides a more realistic picture of a person’s life as compared to a scripted lab data collection which constrains users to a few basic activities. A system developed with data collected in lab settings fails to capture intrinsic behaviors in every day in-the-wild conditions. The data collection protocol is described in detail in [8], and we provide a brief summary in this section. The data is collected from sixty users with their personal devices using specifically designed applications for Android, iPhone, and Pebble-watch unit. Every minute an app collected 20 s of measurements from multiple sensors and asked the user to provide multiple labels that define their environment, behavior, and activities from a selection of 100 contextual labels. In total, the dataset consists of 300,000+ labeled and unlabeled measurements of various heterogeneous sensors. We utilize pre-computed features from six modalities: phone-accelerometer (Acc), phone-gyroscope (Gyro), phone-audio (Aud), phone-location (Loc), phone-state (PS), and watch-accelerometer (WAcc). Among Loc features, we only use quick location features (such as user movement) and discard absolute location as it is place specific. By adding features from each sensing modality, we end up with 166 features, where we utilize 51 processed labels provided in the original dataset.

This dataset also naturally highlights the inevitable problem of missing data in real-world studies. For instance, the participants turned off the location service to avoid battery drain, did not wear the smartwatch continuously and sensor malfunction or other factors resulted in missing samples. In this case, even though labels and signals from other modalities are available but instances with missing features cannot be directly used to train a classifier or to make a prediction in the production setting. This either requires imputation or leads to the discarding of expensive-to-obtain labeled data. From 300 k+ instances in the dataset, approximately half of them have all the features available and the rest even though labeled cannot be utilized due to missing values. Therefore, an efficient technique is required to approximate missing data and prevent valuable information from going to waste during learning a context classifier. Similarly, the data collected in-the-wild often have imperfect and imbalanced classes as some of the labels occur only a few times. It can also be attributed to the difference between participants’ routines or their privacy concerns as some classes are entirely missing from their dataset. Hence, learning from imbalanced classes in a principled way becomes crucial to correctly identify true positives. In summary, the *ExtraSensory Dataset* highlights several challenges for context recognition in real-life conditions, including complex behavioral activities, unrestrained personal device usage, and natural environments with habitual routines.

## 4. Methodology

### 4.1. Autoencoder

**Figure 2:**
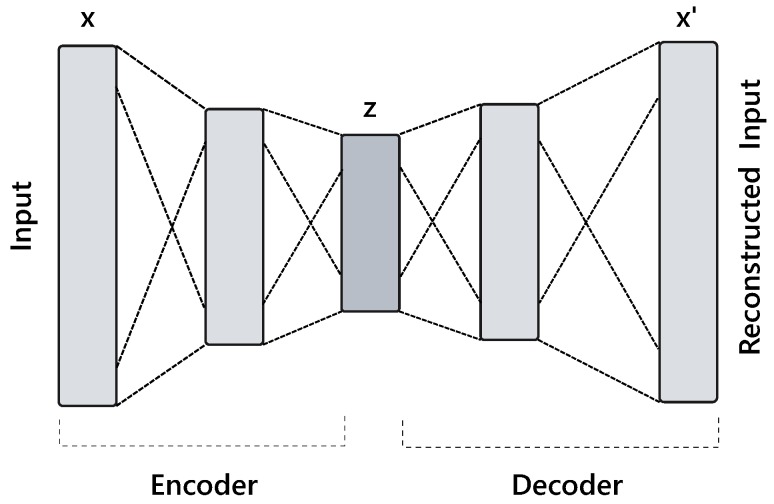
Illustration of an autoencoder network.

An autoencoder is an unsupervised representation learning technique in which a deep neural network is trained to reconstruct its own input x such that the difference between x and the network’s output $x^{\prime }$ is minimized. Briefly, it performs two transformations−encoding $f_{\theta }\left(x\right):R^{n}\to R^{d}$ and decoding $g_{\theta }\left(z\right):R^{d}\to R^{n}$ through deterministic mapping functions, namely, *encoder* and *decoder*. An encoder transforms input vector x to a latent code z, where, a decoder maps the latent representation z to produce an approximation of x. For a single layer neural network these functions can be written as:

(1) $$f_{\theta }\left(x\right):z=\sigma \left(W_{e}x+b_{e}\right),$$

(2) $$g_{\theta^{\prime }}\left(z\right):x^{\prime }=\sigma \left(W_{d}z+b_{d}\right),$$

parameterized by $\theta =\{W_{e},b_{e}\}$ and $\theta^{\prime }=\left\{W_{d},b_{d}\right\}$, where σ is a non-linear activation function (e.g., rectified linear unit), *W* represents a weight coefficient matrix and *b* is a bias vector. The model weights are sometimes tied for regularization such that $W_{d}=W_{e}^{T}$. Figure 2 provides graphical illustration of an autoencoder.

Learning an autoencoder is an effective approach to perform dimensionality reduction and can be thought of as a strict generalization of PCA. Specifically, a 1-layer encoder with linear activation and mean squared error (MSE) loss (see Equation (3)) should be able to learn PCA transformation [38]. Nonetheless, deep models with several hidden layers and non-linear activation functions can learn better high-level and disentangled features from the original input data.

(3) $$L_{MSE}\left(X,X^{\prime }\right)={∥X-X^{\prime }∥}^{2}.$$

The classical autoencoder can be extended in several ways (see for a review [11]). For handling missing input data, a compelling strategy is to train an autoencoder with artificially corrupted input $\tilde{x}$, which acts as an implicit regularization. Usually, the considered corruption includes isotropic Gaussian noise, salt and pepper noise and masking (setting randomly chosen features to zero) [13]. In this case, a network learns to reconstruct a noise-free version $x^{\prime }$ from $\tilde{x}$. Formally, the DAE is trained with stochastic gradient descent to optimize the following objective function:

(4) $$J_{DAE}=\min_{\theta }E_{X}\left[L\left(x,g_{\theta^{\prime }}\left(f_{\theta }\left(\tilde{x}\right)\right)\right)\right],$$

where L represents a loss function like squared error or binary cross entropy.

### 4.2. Adversarial Autoencoder

The Adversarial Autoencoder (AAE) [15] combines adversarial learning [14] with classical autoencoders so it can be used for both learning data embedding and generating synthetic samples. The Generative Adversarial Network (GAN) introduced a novel framework for developing generative models by simultaneously training two networks: (a) the generator *G*, it learns the training instances’ distribution to produce new samples emulating the original samples; and (b) the discriminator network *D*, which differentiates between original and generated samples. Hence, this formulation can be seen as a minimax game between *G* and *D* as shown in Equation (5), where z represents a randomly sampled vector from a certain distribution p(z) (e.g., Gaussian), and x is a sample from the empirical data distribution $p_{data}\left(x\right)$ i.e., training data.

(5) $$\min_{G}\max_{D}E_{X∼p_{data}}\left[\log D\left(x\right)\right]+E_{z∼p\left(z\right)}\left[\log\left(1-D\left(G\left(z\right)\right)\right)\right].$$

**Figure 3:**
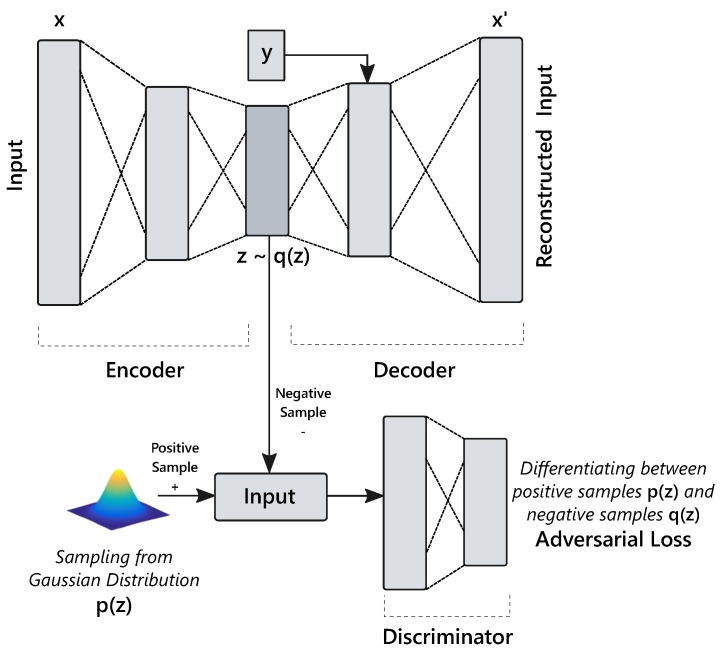
An Adversarial autoencoder network [15].

In AAE, an additional discriminator network is added to an existing autoencoder (see Figure 2) architecture to force the encoder output q(z|x) to match a specific target distribution p(z) as depicted in Figure 3; hence enabling the decoder to act as a generative model. Its training procedure consists of three sequential steps:

- The encoder and decoder networks are trained simultaneously to minimize the reconstruction objective (see Equation (6)). Additionally, the class label information with latent code z can also be provided to the decoder as supervision. Thus, the decoder then uses both z and label information y to reconstruct the input. In addition, conditioning over y enables the decoder to produce class conditional samples.
  (6) $$J_{AE}=\min_{\theta }E_{X}\left[L\left(x,g_{\theta }\left(f_{\theta }\left(x\right)\right)\right)\right].$$
- The discriminator network is then trained to distinguish between true samples from a prior distribution and fake data points (z) generated by an encoder.
- Subsequently, the encoder, whose goal is to deceive the discriminator by minimizing a separate loss function, is updated.

### 4.3. Context Classification

The context recognition under consideration is a multi-label classification problem, where a user’s context at any particular time can be described by a combination of various labels. For instance, a person might be in a meeting, indoor, and with a phone on a table. Formally, it can be defined as follows: $X\in I\;R^{n}$ (i.e., a design matrix) is a set of *m* instances each being *n*-dimensional feature vector having a set of labels *L*. Every instance vector x∈X has a corresponding subset of *L* labels, also called relevant labels; other labels might be missing or can be considered irrelevant for the particular example [24,39]. The goal of the learner is to find a mapping function $f_{c}:x^{n}\to {\left\{0,1\right\}}^{L}$ that assigns labels to an instance. Alternatively, the model predicts a one-hot encoded vector $y\in {\left\{0,1\right\}}^{L}$, where, $y_{i}=1$ (i.e., each element in y) indicates the label is suitable and $y_{i}=0$ represents inapplicability.

The feed-forward neural network can be directly used for multi-label classification with sigmoid activation function in the last layer and binary cross-entropy loss (see Equation (7)); as it is assumed that each label has an equal probability of being selected independently of others. Thus, the binary predictions are acquired by thresholding the continuous output at 0.5.

(7) $$L_{CE}\left(\hat{y},y\right)=-\left[\left(y\log\left(\hat{y}\right)+\left(1-y\right)\log\left(1-\hat{y}\right)\right)\right].$$

As mentioned earlier that in real-world datasets the available contextual labels for each instance could be very sparse (i.e., few $y_{i}=1$). It may happen as, during data collection phase, a user might quickly select a few relevant labels and overlook or intentionally not provide other related labels about the context. In such a setting, just considering an absence of labels as irrelevant may introduce bias in the model, and simply discarding the instance without complete label information limits the opportunity to learn from the available states. Moreover, the positive labels could be very few with a large number of negatives, resulting in an imbalanced dataset. To tackle these issues, we employ a similar instance weighting strategy to [24] while learning a multi-label classifier. In this situation the objective function becomes:

(8) $$J_{C}=\frac{1}{NC}\sum_{i=1}^{N}\sum_{c=1}^{C}\left(\Psi_{i,c}\cdot L_{CE}\left({\hat{y}}_{i,c},y_{i,c}\right)\right),$$

where $L_{ce}$ is the binary cross-entropy loss, and Ψ is an instance-weighting matrix of size *N* × *C* (i.e., number of training examples and total labels, respectively). The instance weights in Ψ are assigned by inverse class frequency. The entries for the missing labels are set to zero, to impose no contribution in the overall cost from such examples.

### 4.4. Model Architecture and Training

The multimodal AAE is developed to alleviate two problems: (a) the likely issue of losing features of the same modality all at once; and (b) synthesizing new labeled samples to increase training dataset size, data augmentation might be helpful to resolve imbalance (in addition to instance weighting), facilitate better understanding of the modeling process, and enable data sharing when original dataset cannot be distributed directly, e.g., due to privacy concerns.

We start the model training process by normalizing continuous features in the range [0,1] with summary statistics calculated from the training set. Next, all the missing features are filled-in with a particular value i.e., −1. It is essential to represent missing data with a distinct value that could not occur in the original. After this minimal pre-processing, a model is trained to reconstruct and synthesize from the clean samples (with all the features available) to provide noise-free ground truth *X*. During reconstruction training, each feature vector x∈X is corrupted with a structured noise [13,29] to get a corrupted version $\tilde{x}$ as (1) masked noise is added to randomly selected 5% of the features; (2) all the features from three or more randomly chosen modalities are set to −1, hence emulating missing data; and (3) dropout is applied. The goal of the autoencoder is then to reproduce clean feature vector x from a noisy version $\tilde{x}$ or in other words to predict reasonably close values of the missing features from the available ones. For example, the model may choose an accelerometer signal from the phone to interpolate smartwatch’s accelerometer features or phone states and accelerometer to approximate location features. Furthermore, for synthesizing novel (class conditional) samples, an independent supervised AAE model is trained without introducing any noise in the input and with a slightly different architecture.

**Figure 4:**
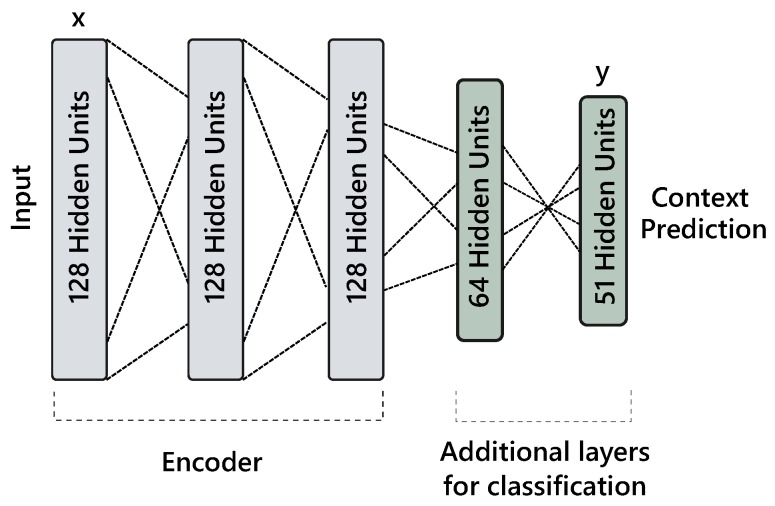
Illustration of an adversarial autoencoder (AAE) based classification network.

After training the AAE model with clean examples for which all sensory modalities are available, it can be extended for multi-label classification. In this situation, either a separate network is learned or additional layers are connected to encoder network to classify a user’s behavioral context (see Figure 4). For latter, the error is backpropagated through the full network; including encoder and classification layers. Moreover, during the classifier training phase, we keep adding noise in the input as mentioned earlier. To leverage the entire dataset for classification, the noisy features are first reconstructed with the learned autoencoder model and combined with the cleaned data. The class weights are calculated from the combined training set (see Section 4.3), where zero weight is assigned to missing labels. Thus, this formulation allows us to learn from any combination of noisy, clean, labeled and unlabeled data.

We employ binary cross-entropy (see Equation (7)) for reconstruction loss rather than MSE as it led to consistently better results in earlier exploration. Since cross-entropy deals with binary values, all the features are first normalized to lie between zero and one as mentioned earlier. We train the reconstruction network in an unsupervised manner, while the synthesizing model is provided with supervision through the decoder network as one-hot encoded vector y of class labels. The missing labels y are simply represented with zeros instead of −1 as we wanted to utilize both labeled and unlabeled instances. The supervision of decoder network also allows the model to better shape the distribution of the hidden code by disentangling label information from compressed representation [15]. Likewise, the samples from Gaussian distribution are provided to a discriminator network as positive examples and hidden code z as negative examples to align the aggregated posterior to match the prior distribution.

To assess the robustness of our approach for filling-in lost sensor features, we compared it with PCA reconstruction by applying inverse transformation to the reduced 75-dimensional principle components vector. In addition, we evaluated multi-label classification performance by utilizing the learned embedding, and training an extended network on top of an encoder and comparing them with four different ways of dealing with the missing data: mean substitution, filling it with a median, replacing missing values with −1, and using a dimensionality reduction method i.e., PCA. To facilitate fair comparison, we limit the reduction of original 166 features to 75-dimensional feature vector, it allows PCA to capture 98% of the variance. We also experimented with a standard DAE model but found it to perform similarly to AEE for feature reconstruction.

The visual fidelity and the supervised classification task are used to examine the quality of the synthetic samples produced by the (decoder) generative model. We train a context classification model on synthetic data and evaluate its performance on the held-out real test set and vice-versa. Because the decoder is trained with supervision it enables us to generate class conditional samples. For generating labeled data, we use labels from the (real) training set and feed it together with the Gaussian noise into the decoder. Another strategy for data augmentation could be to first sample class labels and then use those for producing synthetic features. However, as we are dealing with multi-label classification, where labels jointly explain the user’s context, arbitrarily sampling them is not feasible as it may lead to inconsistent behaviors and activities (such as, sleeping during running). Therefore, we straightforwardly utilize the clean training set labels to sample synthetic data points.

### 4.5. Implementation

Our approach is implemented in Tensorflow [40]. We initialized the weights with Xavier [41] technique and biases with zeros. We use Adam [42] optimizer with fixed but different learning rates for reconstruction and synthesizing models. For the former, the learning rates of $3e^{-4}$, $5e^{-4}$ and $5e^{-4}$ are used for adversarial and reconstruction and classification losses, respectively. While in the latter, $1e^{-3}$, $1e^{-3}$ and $5e^{-4}$ are used for reconstruction, adversarial and classification losses, respectively. We employ l2-regularization on encoder’s and classifier’s weights with a rate of $1e^{-5}$. The rest of the hyper-parameters are minimally tuned on the (internal) validation set by dividing the training folds data into a ratio of 80–20 to discover a architecture that gives optimal performance across users. The suitable configuration of reconstruction network is found to be 3 layers encoder and decoder with 128 hidden units in each layer and dropout [12] with a rate of 0.2 on the input layer. The classification network contains a single hidden layer with 64 units. Similarly, the synthesizing model contains 2 hidden layers with 128 and 10 units and dropout of 0.2 is applied on encoding layer z. However, during sampling from the decoder network, we apply dropout with 0.75. The LeakyReLU activation is used in all the layers except for the classifier trained on synthetic data, where ReLU performed better. Moreover, we also experimented with several batch sizes and found 64 to produce optimal results. We train the models for a maximum of 30 epochs and utilize early-stopping to save the model based on internal validation set performance.

### 4.6. Performance Evaluation

**Figure 5:**
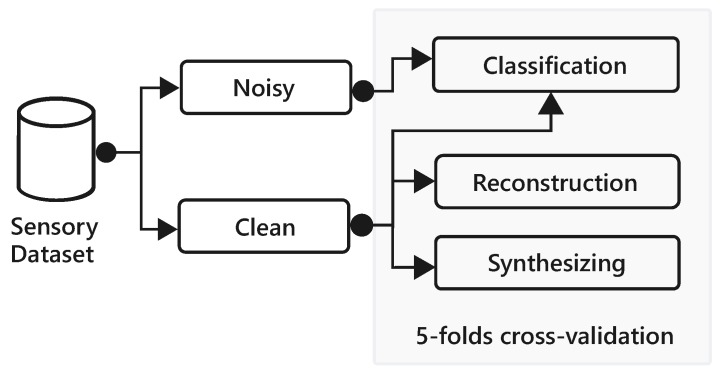
Data split for reconstruction, synthesizing, and classification experiments.

We evaluate reconstruction and classification performance through five-folds cross-validation, where each fold has 48 users for training and 12 users for testing; with the same folds as of [8]. The cross-validation technique is used to show the robustness of our approach when the entire data of users are held-out as test-set during experiments. For hyper-parameters optimization in this setting, we randomly divide a training set into 80% training and 20% internal validation set. The same approach is employed to evaluate the quality of synthetic data points via a supervised classification task. Figure 5 depicts the data division for imputation and classification experiments. The entire dataset is first split-up into clean and noisy parts, where clean data is used for training and measuring the performance of restoring missing features as described in Section 4.4. The noisy data is then interpolated using a learned model and combined with the clean version to use for context classification task. However, we use only clean data to train and evaluate the synthesizing model, the artificial data generated from the AAE is used to train a classifier and its performance is evaluated on real test (folds) data.

The performance of approximating missing data is measured with root mean square error (RMSE) as:

(9) $$\mathrm{RMSE}=\sqrt{E[{\left(X-\tilde{X}\right)}^{2}]}.$$

The multi-label classification is evaluated through balanced accuracy (BA) derived from sensitivity (or recall) and specificity (or true negative rate) as shown in Equation (10). BA is a more robust and fair measure of performance for imbalanced data as it is not sensitive to class skew as opposed to average accuracy, precision and f-score which can over or under emphasize the rare labels [24]. Likewise, it is important to note that, the evaluation metrics are calculated independently for each label of the 51 labels and averaged afterwards.

(10) $$\begin{array}{cc} \mathrm{Sensitivity} & =tp/(tp+fn),\\ \mathrm{Specificity} & =tn/(tn+fp),\\ \mathrm{Balanced}\;\mathrm{Accuracy} & =(\mathrm{Sensitivity}+\mathrm{Specificity})/2. \end{array}$$

## 5. Experimental Results

### 5.1. Modality Reconstruction

We first seek to validate the capability of the AAE network to restore the missing modalities. It is evaluated in comparison with PCA reconstruction, which is achieved by projecting the original 166 features onto a lower dimensional space, having a feature vector of length 75 and then applying an inverse transformation on it to get the original data space. The PCA is able to capture 98% of the variance in the clean training data and thus to set a reasonably strong baseline. However, the AAE network trained with structured noise significantly outperformed the PCA reconstruction by achieving an average RMSE of 0.227 compared with 0.937 on the clean subset of the test folds. To assess the performance of the reconstruction of all the features of each data source, the entire modality is dropped and restored with both procedures. Table 1 provides RMSE averaged across folds and number of features for each modality used from the original dataset. Apart from location features, the AAE network outperforms PCA on the reconstruction of every modality. For gyroscope, we noticed a performance drop on test set of fold 4 which can be due to relatively fewer number of instances from the participants in the testing fold. The reason for comparatively lower performance on the phone state can be attributed to these features being binary and cannot be perfectly approximated with continuous functions.

**Figure 6:**
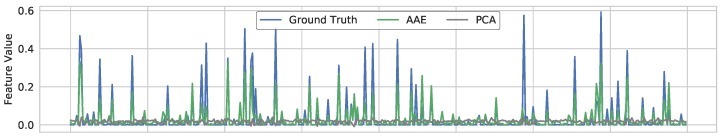
Restoration of an (phone) accelerometer feature values with the AAE and PCA. The entire modality is dropped and reconstructed using features from the remaining signals.

**Figure 7:**
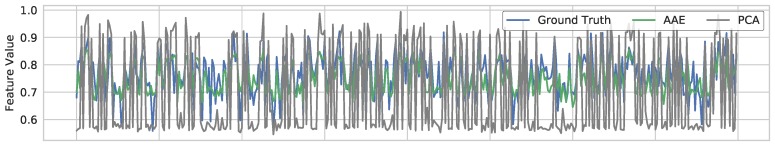
Restoration of an audio (MFCC) feature values with AAE and PCA. An entire modality is dropped and reconstructed using features from the remaining signals.

The AAE is able to learn compressed non-linear representations that are sufficient to capture the correlation between different features. Hence, it provides a close approximation of the features from the lost modality through leveraging the available signals. Figure 6 illustrates this point, where an accelerometer signal (from phone) is dropped (mimicking a missing signal) and all of its 26 features are reconstructed by leveraging the rest of the modalities. The AAE network predicted very realistic values of the missing features that are masked with special value −1. On the contrary, the PCA restoration is stuck around values near zero; failing to capture the feature variance. We think, it could be because PCA does a linear transformation, while the features may have an inherent non-linear relationship that can be extracted well using autoencoders. The difference between the considered methods is also apparent in Figure 7 for filling-in values of features extracted from an audio signal. Here, PCA fluctuates between zero and one, failing to recover the values, whereas, AAE largely recovers values that are close to the ground truth.

### 5.2. Classification with Adversarial Autoencode Representations

**Figure 8:**
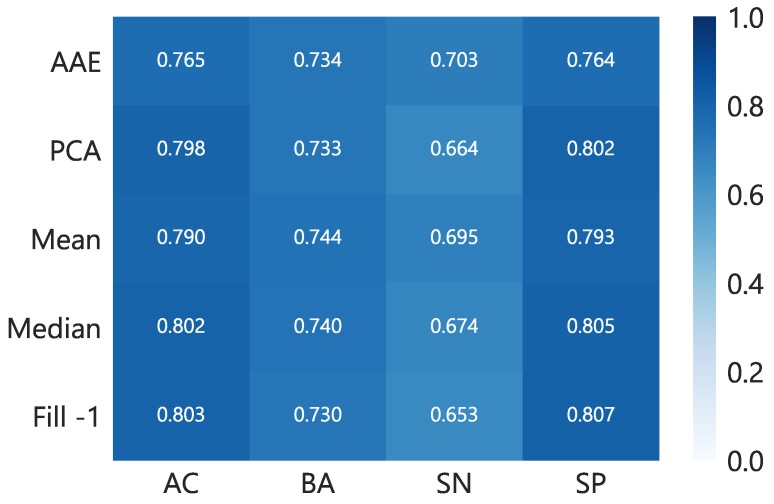
Classification results of 5-folds cross-validation with combined clean and reconstructed noisy data. This resembles the situation when all the modalities are available during learning and inference phases. We notice the AAE network performs better than other technique with high recall rate of 0.703. AC, BA, SN, and SP stand for accuracy, balanced accuracy, sensitivity, and specificity, respectively.

In order to test the ability of AAE to learn a latent code irrespective of missing modalities, we also performed classification experiments with combined, noisy and clean datasets. The feature vector x is passed into the learned autoencoder to get a compressed representation z of 128 dimensions. This embedding is used to train a 1-layer neural network and compared with other methods of missing data imputation such as filling with mean, median or −1 and a dimensionality reduction technique i.e., PCA. Figure 8 provides results on various metrics for cross-validation using considered procedures. We did not find a significant difference between the classifiers trained on embedding and other methods. However, the recall (sensitivity) of AAE is found to be better but somewhat close to the mean imputation. The results obtained here are in line with [29] that used an encoded representation for mood prediction and found no improvement. Similarly, in our case, the reason for unchanged performance could be that a large part of the data is clean and the extracted features are based on extensive domain-knowledge which are highly discriminative. Nevertheless, the latent encoding acquired via AAE can be seen as privacy-preserving representation of otherwise sensitive personal data. Moreover, if an autoencoder is trained with recent advancements made in combining deep models with differential privacy [43], even stronger privacy guarantee can be provided.

### 5.3. Context Recognition with Several Missing Modalities

For better assessment of AAE capability to handle missing data, we simulated multiple scenarios where several modalities are lost at once. These experiments reasonably mimic a real-world situation for the classifier in which a user may turn-off the location service, forget to wear a smartwatch or may be taking a call (such that the audio modality is missing). Thus, as a baseline, we employ techniques to handle missing data through dimensionality reduction and imputation as described earlier and train a classification model with the same configuration (see Section 4.4). The AAE model is extended by adding a classifier network on top of an encoder to directly make predictions for the user context, as explained in Section 4.4.

**Figure 9:**
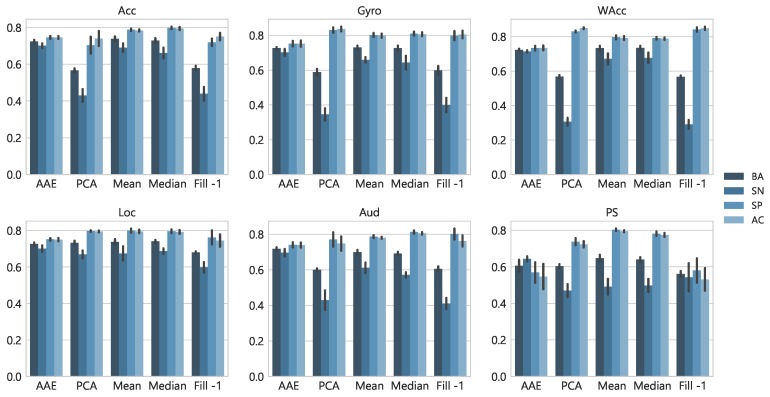
Average evaluation metrics for 51 contextual labels with 5-folds cross-validation. All the features from the corresponding modality are dropped and imputed with all the considered techniques.

We begin by investigating the effect of losing each of the six modalities one by one on the classification performance. Figure 9 summarizes the classification results by utilizing different techniques to handle missing features. The classifier learned through extending the AAE network persistently achieved superior performance compared to the others as can be seen from high BA and true positive rate.

**Figure 10:**
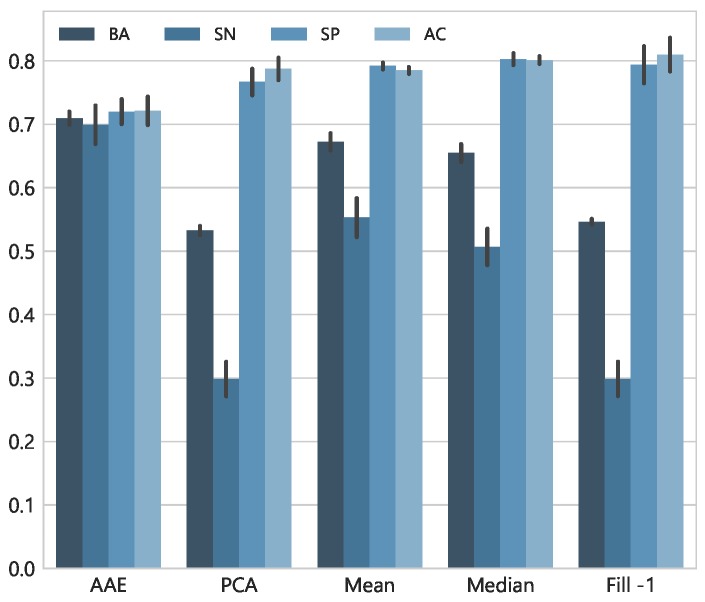
Average evaluation metrics for 51 contextual labels with 5-folds cross-validation. All the features from *Acc*, *Gyro* and *Aud* modalities are dropped and restored with a specific technique.

**Figure 11:**
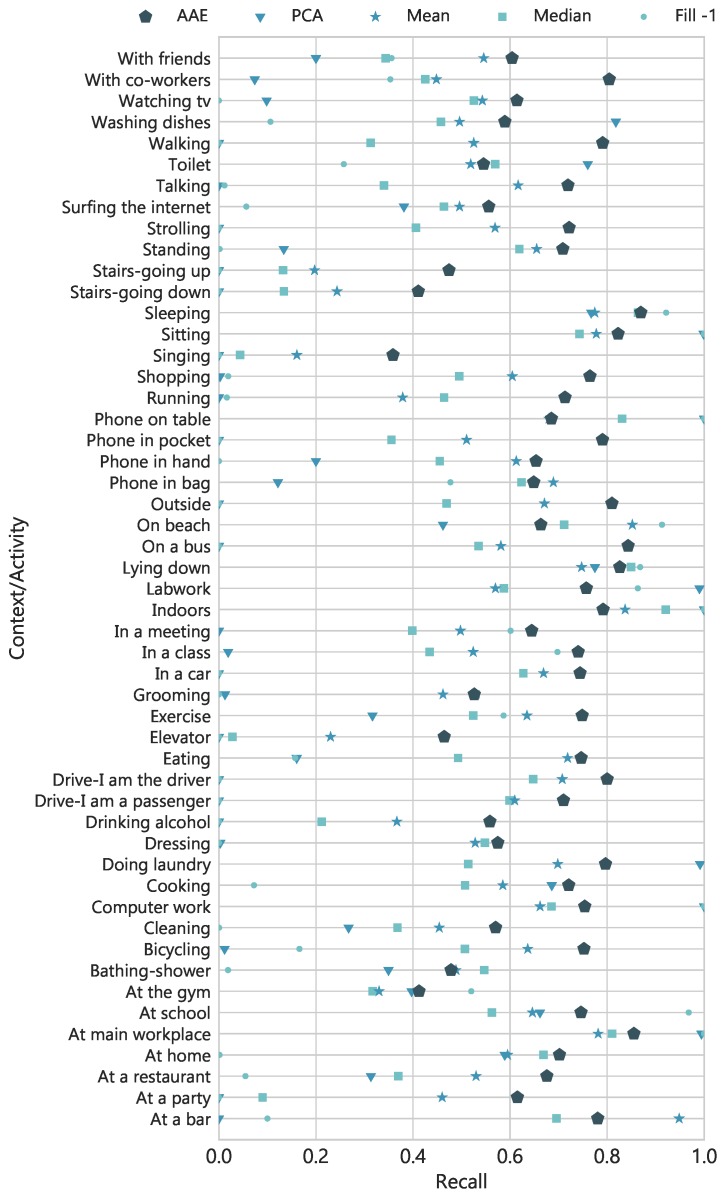
Recall of 51 contextual labels with 5-folds cross-validation. All the features from *Acc*, *Gyro* and *Aud* modalities are dropped to emulate missing features and imputed with different techniques to train a classifier.

Next, we experimented with dropping three important signals i.e., *Acc*, *Gyro*, and *Aud* at once. Figure 10 shows the averaged results across labels and testing folds, when entire feature vectors of the considered modalities are restored with each method. The simplest technique of filling-in missing data with −1 performed poorly with the lowest recall rate and the same goes for PCA which fails to restore the values. However, mean and median imputation performed moderately better as compared to the these two. The AAE achieved better BA and recall rate of 0.710 and 0.700, respectively. It is important to note that the data is highly imbalanced with few positive samples. Therefore, only considering naïve accuracy or true negative rate provides an incomplete picture of the models’ performance. Moreover, to see the fine differences between true positive rates of each technique, Figure 11 presents recall rate for all 51 contextual labels. Overall, the AAE network showed superior results across the labels, highlighting its predictive power to very well handle the noisy inputs.

Next, we evaluated a scenario when four modalities, namely, *Gyro*, *WAcc*, *Loc* and *Aud* are missing together. Specifically, these sensors have high chances of not being available in real-world conditions. Table 2a provides results of the experiment, as earlier, the traditional imputation procedures failed to account for the correct identification of true positives. The AAE gracefully handles missing values with BA of 0.713; through learning important characteristics of data distribution on the training set. Likewise, we tested another scenario with only *WAcc*, *Loc* and *Aud* being missing. Table 2b shows that AAE maintained BA at 0.723 even when nearly half of the features from three important modalities are missing. We further assess the classifier’s behavior, in a case when a user does not provide access to location service and does not wear a smartwatch, i.e., *WAcc* and *Loc* are not available. Table 2c provides these results and indicates that mean/median imputations and AAE showed similar performance on BA metric but the AAE has the highest recall rate of 0.704 among the rest. It highlights the consistent predictive power of AAE based classification network for real-world context recognition applications. Moreover, regardless of the number of missing modalities, the AAE performed superior as compared to other classical ways to handle the lost data.

### 5.4. Generating Realistic Multimodal Data

One of the key goals of this paper is to build a model capable of producing realistic data points and especially features extracted from sensory data. To demonstrate the ability of AAE to generate synthetic data, we evaluate its performance through visual fidelity and classification. The data generated by the AAE is used to train a classifier, which is then tested on real data instances. Similarly, a model is also trained on real data and evaluated on synthetic test data generated by the AAE. This requires the artificial data to have labels, we can provide these labels to the decoder (generator) as supervision, either by sampling them independently or by an additional network (added to an AAE) predict these class labels. Here, we utilized (the former method) using training or test set labels to generate the data, as applicable. This metric of evaluation is also more suitable compared to visual analysis as it determines the ability of synthetic data to be used for real applications. The results of the classification experiments are presented in Table 3, which compares the performance achieved for multi-label context recognition with real and artificial data. It can be seen that the model trained on synthetically generated data achieved close results (BA of 0.715 vs. 0.752) as of when a model is learned on an original data. Likewise, the performance is also optimal (BA of 0.700) when synthetic test data generated using test set labels and random noise are assessed on a classifier learned with real samples.

**Figure 12:**
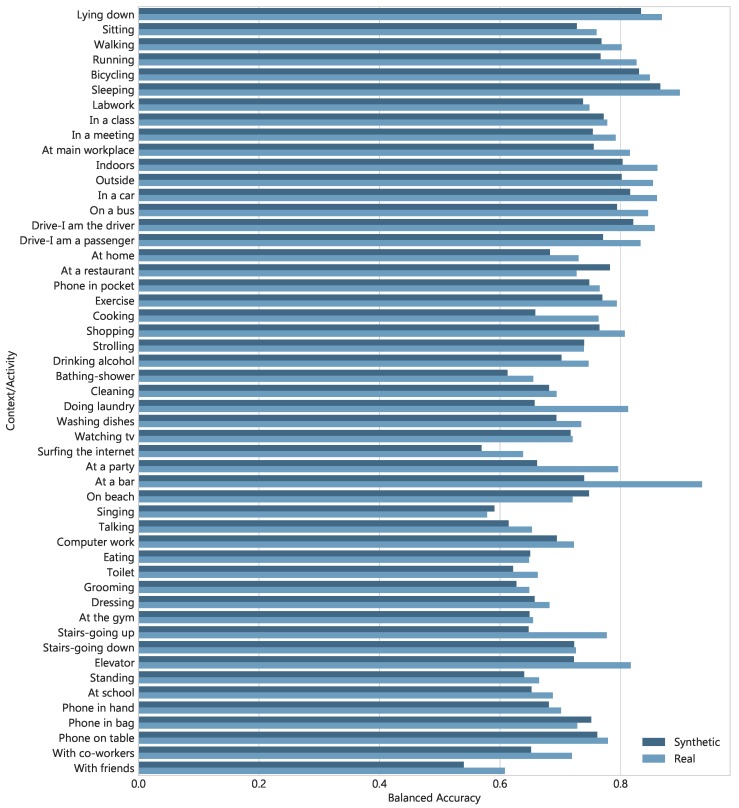
Obtained balanced accuracy of 51 contextual labels for two classifiers trained with real and synthetic samples–evaluation is done on real test data with 5-folds cross-validation.

**Figure 13:**
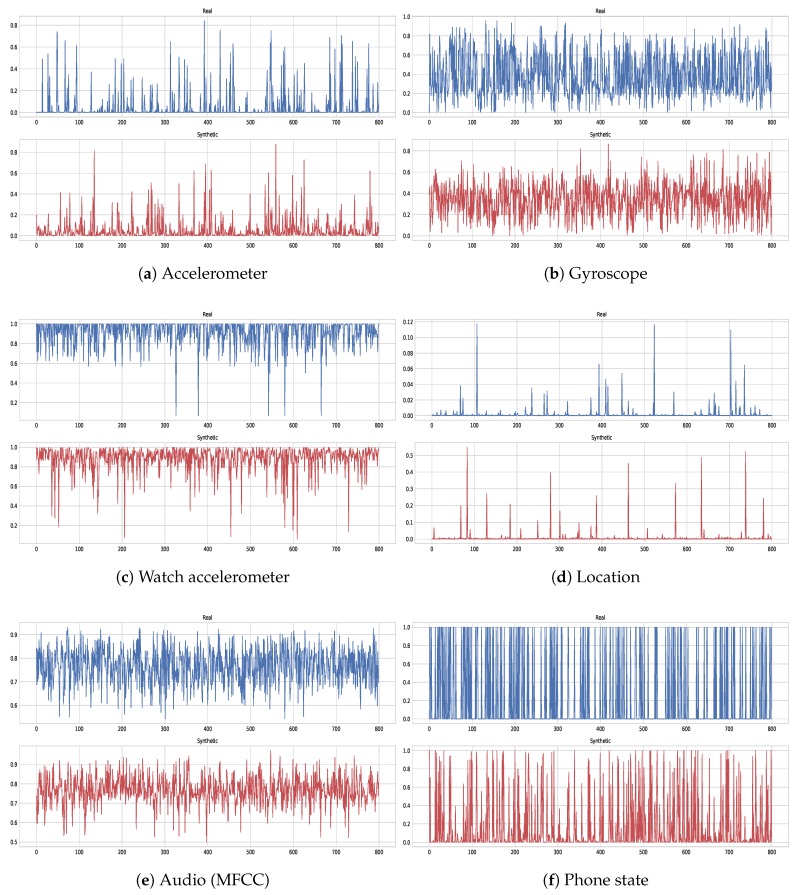
Examples of real (blue, **top**) and generated (red, **bottom**) samples of a randomly selected feature with AAE.

To get a better appreciation of these results, Figure 12 provides BA of each class label for models trained on real and synthetic instances− evaluated on a real test set. We notice that, for some class labels the BA score is equal to or larger than the model learned with real data, such as for classes: *Phone in bag*, *Singing*, *On beach*, and *At a restaurant*. It indicates that the AAE generates realistic enough samples to train a classifier which then achieves high performance on real test data. Furthermore, we also validate the quality of generated samples by visual inspection. It is helpful as we can see from the generated samples if they have the similar characteristics and dynamics as the one we wish to model. Figure 13 illustrates both real and generated examples, the essential thing to notice is that real and synthetic values exhibit similar shift, peaks, and local correlations that are captured well by the AAE. However, binary (discrete) features belonging to phone states such as, is phone connected to Wi-Fi etc. are hard to perfectly reconstruct but they can be easily binarized by thresholding at a particular value.

## 6. Discussion and Conclusions

We proposed a method utilizing an AAE for synthesizing and restoring missing sensory data to facilitate user context detection. The signals loss commonly happens during real-world data collection and in realistic situations after model deployment in-the-wild. For example, a user may prefer to not wear a smartwatch, hence, no signals (or features) from a smartwatch that are used during development will be available for inference. Our empirical results demonstrate that the AAE network trained with structured noise can provide a realistic reconstruction of features from the lost modalities as compared to other methods, such as PCA. Similarly, we show the AAE model trained with supervision to a decoder network produce realistic synthetic data, which further can be used for real applications. We have shown the data generation capability of our network through visual fidelity analysis and by comparing classification performance with real data. In the latter, we do training on the artificial data and evaluation of real instances, and training on real and validation on synthetic samples. This methodology allows researchers to develop robust models that are able to learn noise invariant representations and inherently handle several missing modalities. It also enables leveraging artificial data to increase training set size, and data sharing which is occasionally not possible due to the sensitive nature of the personal data.

The presented network has several other advantages, it allows to utilize an entire dataset for learning i.e., any combination of labeled, unlabeled noisy and clean instances. We see a consistent performance of our classifier trained by extending the encoder network, even when several modalities (i.e., more than half of the features) are dropped to emulate missing sensors. Broadly, unlike prior methods for handling missing input data, where a model failed to detect true positive correctly, AAE maintains its ability to recognize user context with high performance. This highlights an important characteristic of the described technique that even if some signals are not available e.g., when users opt-out of location service or do not wear a smartwatch, still their partial data can be used to get accurate predictions. Besides, the model developed with the proposed technique could be a very attractive feature for users concerned about their privacy concerns regarding location data. Likewise, a classifier trained on embedding provides similar performance as the original feature set, which means raw features would not have to be stored and can be shared with other researchers while preserving users’ privacy [29]. The privacy guarantee can be further enhanced by taking advantage of recent advances made in combining deep learning with differential privacy [43].

We notice that labels reported by the users are sparse, resulting in an imbalanced dataset. To deal with this, an instance weighting strategy same as in [24] is applied. Although, we experimented with resolving imbalance through synthetic data only but results were not satisfactory (unless combined with instance weighting); we believe this requires further exploration. Likewise, AAE can be extended to do semi-supervised learning taking advantage of unlabeled examples. It can further help in the collection of a large dataset with a low mental load for the user as it reduces the need for labeling every example. Another area of potential improvement could be an ensemble of multi-layer neural networks efficiently compressed to do real-time detection on an edge device with minimum resource utilization.

## Figures and Tables

**Table 1:** Root mean square error (RMSE) for each modality averaged over 5-folds cross-validation.

Modality	# of Features	PCA	AAE
Accelerometer (Acc)	26	1.104 ± 0.075	0.104 ± 0.016
Gyroscope (Gyro)	26	1.423 ± 0.967	0.686 ± 1.291
WAccelerometer (WAcc)	46	1.257 ± 0.007	0.147 ± 0.003
Location (Loc)	6	0.009 ± 0.003	0.009 ± 0.003
Audio (Aud)	28	1.255 ± 0.015	0.080 ± 0.006
Phone State (PS)	34	0.578 ± 0.000	0.337 ± 0.011

PCA: principal component analysis.

**Table 2:** Classification results for 5-folds cross-validation with different missing modalities that are restored with a specific method. The reported metrics are averaged over 51 labels and BA stands for balanced accuracy.

(a) Missing: *Gyro*, *WAcc*, *Loc* and *Aud*				
	**BA**	**Sensitivity**	**Specificity**	**Accuracy**
AAE	0.713 ± 0.008	0.711 ± 0.021	0.716 ± 0.021	0.716 ± 0.024
PCA	0.526 ± 0.007	0.249 ± 0.040	0.802 ± 0.041	0.825 ± 0.034
Mean	0.669 ± 0.023	0.548 ± 0.056	0.791 ± 0.025	0.785 ± 0.022
Median	0.657 ± 0.015	0.502 ± 0.045	0.812 ± 0.022	0.808 ± 0.017
Fill -1	0.519 ± 0.004	0.175 ± 0.012	0.862 ± 0.004	0.857 ± 0.013
(**b**) Missing: *WAcc*, *Loc* and *Aud*				
	**BA**	**Sensitivity**	**Specificity**	**Accuracy**
AAE	0.723 ± 0.007	0.729 ± 0.017	0.718 ± 0.013	0.721 ± 0.014
PCA	0.549 ± 0.02	0.255 ± 0.052	0.842 ± 0.013	0.847 ± 0.019
Mean	0.682 ± 0.017	0.567 ± 0.04	0.797 ± 0.014	0.79 ± 0.014
Median	0.678 ± 0.014	0.543 ± 0.028	0.814 ± 0.005	0.806 ± 0.004
Fill -1	0.547 ± 0.016	0.209 ± 0.087	0.885 ± 0.055	0.836 ± 0.047
(**c**) Missing: *WAcc* and *Loc*				
	**BA**	**Sensitivity**	**Specificity**	**Accuracy**
AAE	0.722 ± 0.010	0.704 ± 0.029	0.74 ± 0.018	0.742 ± 0.020
PCA	0.568 ± 0.012	0.300 ± 0.038	0.835 ± 0.016	0.856 ± 0.010
Mean	0.735 ± 0.011	0.678 ± 0.028	0.793 ± 0.009	0.789 ± 0.008
Median	0.727 ± 0.012	0.653 ± 0.035	0.801 ± 0.020	0.796 ± 0.020
Fill -1	0.564 ± 0.026	0.270 ± 0.064	0.859 ± 0.012	0.840 ± 0.008

**Table 3:** Performance of 1-layer neural network for context recognition when: (a) both the training and the test sets are real (Real, first row); (b) a model trained with synthetic data and the test set is real (TSTR, second row); and (c) the training set is real and the test set is synthetic (TRTS, bottom row).

	BA	Sensitivity	Specificity	Accuracy
Real	0.753 ± 0.011	0.762 ± 0.014	0.745 ± 0.016	0.749 ± 0.015
TSTR	0.715 ± 0.011	0.731 ± 0.035	0.700 ± 0.036	0.705 ± 0.034
TRTS	0.700 ± 0.020	0.656 ± 0.035	0.744 ± 0.033	0.744 ± 0.030

TSTR: Training on synthetic and testing on real

## References

[1] Rashidi P., Mihailidis A. (2013). A survey on ambient-assisted living tools for older adults. IEEE J. Biomed. Health Inform..

[2] Nahum-Shani I., Smith S.N., Tewari A., Witkiewitz K., Collins L.M., Spring B., Murphy S. (2014). Just in Time Adaptive Interventions (JITAIs): An Organizing Framework for Ongoing Health Behavior Support.

[3] Avci A., Bosch S., Marin-Perianu M., Marin-Perianu R., Havinga P. Activity recognition using inertial sensing for healthcare, wellbeing and sports applications: A survey. Proceedings of the 23th International Conference on Architecture of Computing Systems.

[4] Rabbi M., Aung M.H., Zhang M., Choudhury T. MyBehavior: Automatic personalized health feedback from user behaviors and preferences using smartphones. Proceedings of the 2015 ACM International Joint Conference on Pervasive and Ubiquitous Computing.

[5] Althoff T., Hicks J.L., King A.C., Delp S.L., Leskovec J. (2017). Large-scale physical activity data reveal worldwide activity inequality. Nature.

[6] Joshua L., Varghese K. (2010). Accelerometer-based activity recognition in construction. J. Comput. Civ. Eng..

[7] Dey A.K., Wac K., Ferreira D., Tassini K., Hong J.H., Ramos J. Getting closer: An empirical investigation of the proximity of user to their smart phones. Proceedings of the 13th International Conference on Ubiquitous Computing.

[8] Vaizman Y., Ellis K., Lanckriet G. (2017). Recognizing Detailed Human Context in the Wild from Smartphones and Smartwatches. IEEE Pervas. Comput..

[9] Kang H. (2013). The prevention and handling of the missing data. Korean J. Anesthesiol..

[10] Gelman A., Hill J. (2006). Missing-data imputation. Data Analysis Using Regression and Multilevel/Hierarchical Models.

[11] Bengio Y., Courville A., Vincent P. (2013). Representation learning: A review and new perspectives. IEEE Trans. Pattern Anal. Mach. Intell..

[12] Srivastava N., Hinton G., Krizhevsky A., Sutskever I., Salakhutdinov R. (2014). Dropout: A simple way to prevent neural networks from overfitting. J. Mach. Learn. Res..

[13] Vincent P., Larochelle H., Lajoie I., Bengio Y., Manzagol P.A. (2010). Stacked denoising autoencoders: Learning useful representations in a deep network with a local denoising criterion. J. Mach. Learn. Res..

[14] Goodfellow I., Pouget-Abadie J., Mirza M., Xu B., Warde-Farley D., Ozair S., Courville A., Bengio Y. (2014). Generative adversarial nets. Advances in Neural Information Processing Systems 27, Proceedings of the Annual Conference on Neural Information Processing Systems, Montreal, QC, Canada, 8–13 December 2014.

[15] Makhzani A., Shlens J., Jaitly N., Goodfellow I., Frey B. (2015). Adversarial autoencoders. arXiv.

[16] Guiry J.J., Van de Ven P., Nelson J. (2014). Multi-sensor fusion for enhanced contextual awareness of everyday activities with ubiquitous devices. Sensors.

[17] Wang A., Chen G., Shang C., Zhang M., Liu L. Human activity recognition in a smart home environment with stacked denoising autoencoders. Proceedings of the International Conference on Web-Age Information Management.

[18] Li Y., Shi D., Ding B., Liu D. (2014). Unsupervised feature learning for human activity recognition using smartphone sensors. Mining Intelligence and Knowledge Exploration.

[19] Plötz T., Hammerla N.Y., Olivier P. Feature learning for activity recognition in ubiquitous computing. Proceedings of the IJCAI Proceedings—International Joint Conference on Artificial Intelligence.

[20] Wang J., Chen Y., Hao S., Peng X., Hu L. (2017). Deep learning for sensor-based activity recognition: A survey. arXiv.

[21] Ding M., Fan G. (2015). Multilayer Joint Gait-Pose Manifolds for Human Gait Motion Modeling. IEEE Trans. Cybern..

[22] Zhang X., Ding M., Fan G. (2017). Video-based human walking estimation using joint gait and pose manifolds. IEEE Trans. Circuits Syst. Video Technol..

[23] Chen C., Jafari R., Kehtarnavaz N. (2017). A survey of depth and inertial sensor fusion for human action recognition. Multimedia Tools Appl..

[24] Vaizman Y., Weibel N., Lanckriet G. (2018). Context Recognition In-the-Wild: Unified Model for Multi-Modal Sensors and Multi-Label Classification. Proc. ACM Interact. Mob. Wearable Ubiquitous Technol..

[25] Thompson B.B., Marks R., El-Sharkawi M.A. On the contractive nature of autoencoders: Application to missing sensor restoration. Proceedings of the International Joint Conference on Neural Networks.

[26] Nelwamondo F.V., Mohamed S., Marwala T. (2007). Missing data: A comparison of neural network and expectation maximization techniques. arXiv.

[27] Duan Y., Lv Y., Kang W., Zhao Y. A deep learning based approach for traffic data imputation. Proceedings of the 17th International IEEE Conference on Intelligent Transportation Systems (ITSC).

[28] Beaulieu-Jones B.K., Moore J.H. (2017). Missing data imputation in the electronic health record using deeply learned autoencoders. Proceedings of the Pacific Symposium on Biocomputing 2017.

[29] Jaques N., Taylor S., Sano A., Picard R. Multimodal Autoencoder: A Deep Learning Approach to Filling in Missing Sensor Data and Enabling Better Mood Prediction. Proceedings of the International Conference on Affective Computing and Intelligent Interaction (ACII).

[30] Li J., Struzik Z., Zhang L., Cichocki A. (2015). Feature learning from incomplete EEG with denoising autoencoder. Neurocomputing.

[31] Miotto R., Li L., Kidd B.A., Dudley J.T. (2016). Deep patient: An unsupervised representation to predict the future of patients from the electronic health records. Sci. Rep..

[32] Martinez H.P., Bengio Y., Yannakakis G.N. (2013). Learning deep physiological models of affect. IEEE Comput. Intell. Mag..

[33] Deng J., Xu X., Zhang Z., Frühholz S., Schuller B. (2017). Universum autoencoder-based domain adaptation for speech emotion recognition. IEEE Signal Process. Lett..

[34] Kuchaiev O., Ginsburg B. (2017). Training Deep AutoEncoders for Collaborative Filtering. arXiv.

[35] Yu L., Zhang W., Wang J., Yu Y. SeqGAN: Sequence Generative Adversarial Nets with Policy Gradient. Proceedings of the AAAI.

[36] Choi E., Biswal S., Malin B., Duke J., Stewart W.F., Sun J. (2017). Generating multi-label discrete electronic health records using generative adversarial networks. arXiv.

[37] Esteban C., Hyland S.L., Rätsch G. (2017). Real-valued (medical) time series generation with recurrent conditional GANs. arXiv.

[38] Hinton G.E., Salakhutdinov R.R. (2006). Reducing the dimensionality of data with neural networks. Science.

[39] Nam J., Kim J., Mencía E.L., Gurevych I., Fürnkranz J. (2014). Large-scale multi-label text classification–revisiting neural networks. Proceedings of the Joint European Conference on Machine Learning and Knowledge Discovery in Databases.

[40] Abadi M., Barham P., Chen J., Chen Z., Davis A., Dean J., Devin M., Ghemawat S., Irving G., Isard M. (2016). TensorFlow: A System for Large-Scale Machine Learning. OSDI.

[41] Glorot X., Bengio Y. Understanding the difficulty of training deep feedforward neural networks. Proceedings of the Thirteenth International Conference on Artificial Intelligence and Statistics.

[42] Kingma D.P., Ba J. (2014). Adam: A method for stochastic optimization. arXiv.

[43] Abadi M., Chu A., Goodfellow I., McMahan H.B., Mironov I., Talwar K., Zhang L. Deep learning with differential privacy. Proceedings of the 2016 ACM SIGSAC Conference on Computer and Communications Security.

